# Social Media and brand loyalty in ophthalmological services

**DOI:** 10.22336/rjo.2021.20

**Published:** 2021

**Authors:** Consuela-Mădălina Gheorghe

**Affiliations:** * Philologist, Authorized translator

According to an article published in 2013 in the International Journal of Information Management, by Michel Laroche et al., entitled “To be or not to be in social media: How brand loyalty is affected by social media?”, there seems to be an emergent debate regarding the activities of brands and companies on Social Media. Some researchers consider that Social Media offer an exclusive opportunity for brands to cultivate their relationships with customers, while others think the opposite. As consequence, a new concept appeared, that of brand community based on Social Media. Considering this concept and the one of brand trust and loyalty, there seems to be an influence on the customer centric model, meaning the relationships between the targeted customer and brand, services, ophthalmological organization, and other customers, and brand loyalty. Thus, it was concluded that brand communities established on Social Media have positive effects on the customer and the services, the brand, and the ophthalmological organization, which have positive effects on brand trust and brand loyalty. 

Brand loyalty can be defined as a behavior of the customer that implies the repeatedly purchase of a product or service offered by the same ophthalmological organization, instead of selecting a similar product or service offered by the competition. Brand loyalty is measured by marketers in terms of brand recognition, brand preference and brand insistence. 

Loyal customers continuously buy products or services from their preferred ophthalmological organizations or brand, regardless of the price. For example, an eccentric person who likes and has been accustomed with Gucci eyeglasses will always choose this brand because it satisfies his/ her needs at all levels. 

Regarding loyalty, there are two types: transactional and emotional. According to The Clarus Blog (www.claruscommerce.com), there is a focus on experiential benefits in the media, but transactional benefits still have an important role. Therefore, transactional loyalty refers to traditional benefits, such as discounts and free shipping/ giveaways, which affect consumer’s behavioral loyalty. Moreover, transactional loyalty seems to be an attractive and necessary element in building brand advocates. At the same time, experiential loyalty increases the member experience and creates sustainable emotional connections between brands and customers. It is a fact that customers seek material benefits along with special treatment, which, most of the time, is expressed in the form of experiences. Experiences lead to emotional loyalty, which is the liaison between transactional and true loyalty. Emotional loyalty is defined by a psychological preference and affective attachment to the brand. 

In the same vein, it has been uncovered that there are two types of loyal customers, who are influenced by four factors: dependability, emotional connection, superiority, and Social Media presence. In order to have loyal customers, brands must prove their superiority compared to the competition. Once the customers become loyal, they will improve the brand’s image by commenting, sharing, liking, or posting brand-related content on social platforms. These actions will lead to lots of followers. 

In conclusion, there are some methods to build and maintain customer loyalty, such as offering discounts, rewarding customers, promoting the rewards program, encouraging referrals, creating a point system, creating a partnership with another company, setting up a subscription service, asking for feedback, asking for reviews, responding to feedback & reviews, paying attention to social sentiment, showing gratitude, using a CRM for loyalty management, personalizing sales & marketing, creating loyalty tiers, engaging customers, maintaining a human connection, making the ophthalmological organization available, prioritizing emotional relationships, speaking the language of the customers, being honest, being flexible, remembering the customers’ special days, offering the customers wish lists, etc. 

With the latest analytics technology, ophthalmological organizations can offer discounts on products or ophthalmological services that customers purchase regularly, as well as products that complement prior purchases. Even if it is for elderly, students, or children, offering a discount is the perfect way to recognize (and retain) key segments of the ophthalmological organization base.

Rewards usually represent a pleasant surprise for the customer. When offering rewards, it is important to include an expiration date, so that ophthalmological customers are encouraged to buy the products or services before the deadline. 

A rewards program should be created, but it should also be made known to the customers, so that they would sign up. 

A friend referral program should be created in order to reward both existent customers and gain new ones. 

Considering the loyalty systems that exist nowadays, ophthalmological organizations can offer various types of rewards on purchases, status level, and other customer features. Some ophthalmological organizations offer their customers the possibility to receive rewards or points with partner companies, thus, partnering with other relevant brands represents another way to boost and improve the image of the ophthalmological organization. 

A subscription-based program is also very important to retain customers on long-term and offers incentives for the customers that they may not receive anywhere else.

Informing customers that their feedback is much appreciated by the ophthalmological organization only proves that the organization is committed to improving products or services in order to better satisfy their needs. 

Reviews represent authentic testimonials from real people. A customer who is unsure will always check reviews to find out other people’s experiences with the ophthalmological organization, and decide if they wish to benefit from the products or services of a certain ophthalmological organization. Moreover, collecting reviews also represents an opportunity to transform happy customers into brand advocates. 

Asking the customers for their opinions is of utmost importance, as it is to answer to the customers’ reviews received by the ophthalmological organization. 

Analyzing social sentiment (public perception) is another way to find out how customers think and feel about the ophthalmological organization, expressed via social media platforms. 

Expressing gratitude to the customers is a key element. In addition, ophthalmological organizations should find a way to thank every customer who has bought a product or a service. Gratitude can be expressed via “Thank you” notes, packaging inserts, holiday cards, birthday cards, etc.

A CRM system helps collecting and storing customer data, such as purchase habits, transaction histories, customer service records, etc. This Customer Relationship Management system helps to better understand customers, in order to personalize their experiences and increase the chances of turning them into loyal customers for the brand.

In addition, personalization of the products and services represents one of the most effective ways to draw attention to the brand. 

More purchases and rewards can be offered by setting up a tiered customer loyalty program. Thus, customers earn points with every visit to the ophthalmological organization, which materialize into discounts. It is a fact that engaged customers are loyal customers. 

As far as one-on-one interactions are concerned, they are very much valued, even if everything is digitized nowadays. 

As already known, the customers can take different and many roads to get to the organization, so it is important that there is “speaker” to represent the brand. 

In the competitive market nowadays, customer satisfaction is not considered the element that makes the difference anymore. The modern customer wishes to have an experiential experience, besides the meeting of his/ her basic needs.

Speaking the customer’s language is an expression that may be taken literally, meaning that it is important that the products or services are translated into the native language of the customers so that they are purchased and, at the same time, offering multilingual services is a good way to demonstrate that the ophthalmological organization values all its customers, no matter their ethnicity. 

Honesty is always the best policy, because a small mistake that is not solved immediately, may lead to data breach, which leads to loosing trust and loyalty of the customers. 

Flexibility is also a key element, which will ensure a positive review of the ophthalmological organization. 

The ophthalmological organization should make sure that the marketing department sends emails or postcards to acknowledge the customers’ special days, such as birthdays, for example. They can even offer special birthday discounts or free products or services to help the cause. 

Last, but not least, there are loyalty programs, whose aim is to remind about customers who need to be given the ability to keep track of their favorite products, such as eyeglasses, sunglasses, contact lens, etc. Giving customers the ability to keep track of their favorite items or create wish lists is another feature that most successful loyalty programs offer. 

**Assist. Prof. Consuela-Mădălina Gheorghe, PhD,** **Philologist, Authorized translator**

